# A child with a novel *DDX3X* variant mimicking cerebral palsy: a case report

**DOI:** 10.1186/s13052-020-00850-3

**Published:** 2020-06-29

**Authors:** Liqin Hu, Xiaoqin Xin, Shaobin Lin, Min Luo, Junkun Chen, Hongsheng Qiu, Li Ma, Jungao Huang

**Affiliations:** 1 Department of Gynaecology and Obstetrics, Ganzhou Maternal and Child Health Hospital, Ganzhou, 341000 Jiangxi Province China; 2 Department of Clinical Laboratory, Ganzhou People’s Hospital, Ganzhou, 341000 Jiangxi Province China; 3 Fetal Medicine Centre, The First Affiliated Hospital of Sun Yat-Sen University, Guangzhou, 510080 Guangdong Province China; 4 Department of Pediatric Neurorehabilitation, Ganzhou Maternal and Child Health Hospital, Ganzhou, 341000 Jiangxi Province China; 5 Department of Medical Genetics, Ganzhou Maternal and Child Health Hospital, Ganzhou, 341000 Jiangxi Province China; 6 Neonatology Department, Ganzhou Maternal and Child Health Hospital, Ganzhou, 341000 Jiangxi Province China

**Keywords:** Cerebral palsy, MRX102, Whole-exome sequencing, Diagnostics

## Abstract

**Background:**

Cerebral palsy (CP) is a non-progressive disorder of movement and posture due to a static insult to the brain. In CP, the depth of investigation is guided by the patients’ medical history and their clinical examination. Magnetic resonance imaging (MRI) has a high yield and is widely used for investigation in CP.

**Case presentation:**

In this paper, we report a novel *DDX3X* variant in a girl afflicted with the X-linked mental retardation-102 (MRX102). The girl had been misdiagnosed with CP in her early life based on a comprehensive clinical evaluation and associated clinical features, such as developmental delay, reduced activities of the arms and legs, and abnormal brain MRI. Subsequently, whole-exome sequencing was applied to better distinguish between CP and actual MRX102 with similar characteristics.

**Conclusions:**

We report on a de novo heterozygous *DDX3X* variant mimicking cerebral palsy and suggest a thorough and conscientious review during diagnosis of CP.

## Background

Cerebral Palsy (CP) is a non-progressive group of developmental disorders of movement and posture caused by a static insult occurring in the brain of the fetus or infant. CP is regarded as the most common cause of physical disability in childhood with a prevalence of approximately 2 per 1000 live births [1]. Motor dysfunction in CP has been classified into three categories: spastic, dyskinetic, and ataxic [2]. CP is associated with medical comorbidities, such as intellectual disability, epilepsy, and visual disturbances, and clinical heterogeneity may exist [3, 4]. During the progress of the diagnosis of CP, a neurological syndrome, a thorough family history and examination was suggested to look for signs of a non-CP diagnosis [5]. The presence of non-motor symptoms such as vision or hearing deficits, cranial nerve dysfunction and epilepsy should be paid more attention to [6]. For the diagnosis of CP, it is believed that MRI is the highest yield investigation method, with almost 89% abnormal MRI among children with CP [7]. Meanwhile, considering that the symptoms and signs of CP change over time, some difficulty may be encountered when diagnosing CP, especially for some of the underlying insult. It is possible that there are some diseases that can be confused with CP, especially for ataxic CP [8].

X-linked mental retardation-102 (MRX102; MIM: 300958) has been described in patients with variants of *DDX3X* at Xp11.4 which encodes a DEAD-box RNA helicase [9, 10]. *DDX3X* plays an important role in transcription, splicing, transport of RNA, and translation, and works as a key regulator in the Wnt/β-catenin signaling pathway [11, 12]. *DDX3X* consists of 17 coding exons and is the most common pathogenic gene on the X chromosome. The inheritance of both X-linked dominant and recessive modes has been observed. De novo variants of *DDX3X* have been identified in 1–3% of females with intellectual disability [13]. MRX102 occurs in infancy and may involve the head, face, ears, mouth, skin, and muscle in affected individuals. Although MRX102 mainly manifests as intellectual disability and dyskinesia, symptoms of autism may accompany it [14]. In some patients with *DDX3X* variants, the disorder may even involve the endocrine glands as precocious puberty has been observed. Here, we report the case of a girl diagnosed with ataxic CP as an infant. Subsequently, genetic investigation via whole-exome sequencing of the family indicated that the patient had MRX102, with identification of a novel variant in *DDX3X*. This case may highlight the need for genetic analysis to determine the underlying cause of a disorder before a diagnosis of CP is made.

## Case presentation

**Fig. 1:**
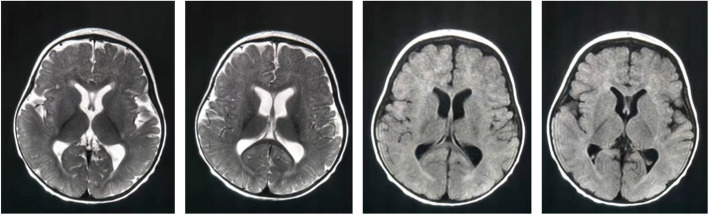
Magnetic resonance imaging scans when the girl was 10 months old show abnormal signals in the posterior horn and trigonometry region of the lateral ventricle; the lateral ventricle and the third ventricle were slightly enlarged

**Fig. 2:**
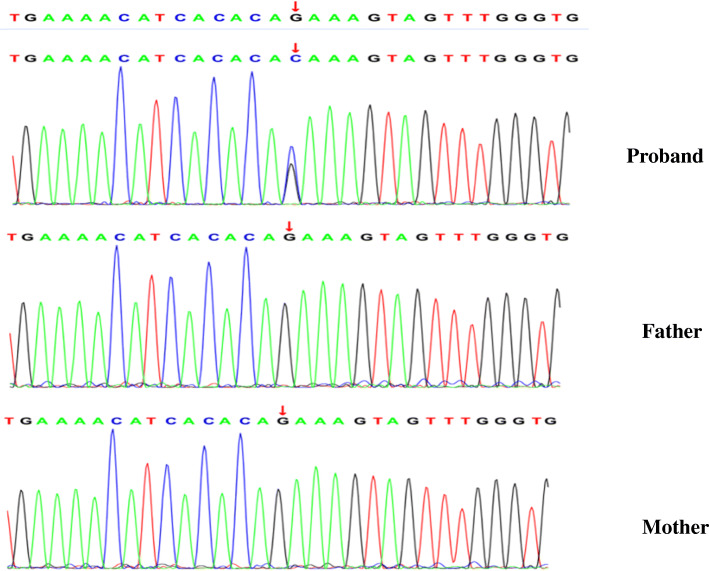
The sequence chromatogram for the mutation: c.1251 G > C located at exon 12 of the *DDX3X* gene in the X chromosome. This mutation existed only in the proband identified by using primers: F (5′-CAGATGCTGGCTCGTGATTT-3′) and R (5′-ACCTGTGAATAACCCCTGCA-3′)

The Chinese girl was born at 39 weeks and 6 days gestation by natural labor following a normal pregnancy when her mother was 27 and her father was 24 years of age. Her birth weight was 3300 g, birth length 52.3 cm, and birth head circumference 34.7 cm. There was no history of asphyxia and abnormalities found in the newborn screening at birth. The girl was unable to sit up by herself at 10 months of age. This developmental delay was documented by pediatricians, along with reduced activities of the arms and legs, and thus a diagnosis of ataxic CP was considered. Brain MRI at that time showed abnormal signals in the posterior horn and trigonometry region of the lateral ventricle, and the lateral and third ventricles were slightly enlarged, which suggested cerebral dysplasia (Fig. 1) At 2 years and 6 months of age, a neurologist noted that the girl had a special face with a prominent forehead, two hollow eye sockets, two low-set ears and a wide bridge of the nose, an unsteady gait and walked with help, fell frequently and was unable to eat with cutlery. In addition, she rarely communicated and had difficulty understanding, bad eye contact, and no reaction upon the calling of her name. There was no history of infection, including hepatitis, tuberculosis, measles, and chicken pox. The tests for the syndromes of Kernig, Brudzinski, and Babinski were negative. Liver, kidney, and heart function were all normal, the level of lactic acid and ammonia in plasma was within limits and the results of the analysis of tandem mass spectrum for peripheral blood were also normal. Considering her chronic non-progressive course with hypotonia and intellectual disability, the girl was diagnosed with ataxic CP. She underwent rehabilitation training between the ages of 1 and 2, but her motor function was still abnormal compared with her peers, especially her intellectual level. Subsequently, genetic analysis was performed with agreement when the patient was 3 years old, and traditional chromosome analysis and chromosome microarray analysis were normal. The girl was then tested with whole-exome sequencing and analysis within her family. The test identified a novel heterozygous variant of the *DDX3X* gene, confirmed by sanger sequencing. The c.1251 G > C variant was located in exon 12 of the *DDX3X* gene, resulting in a p.Gln417His substitution (Fig. 2). This variant was within the functional domain, without benign variants in the clinvar database. The variant was absent from the Exome Aggregation Consortium database (ExAC) and predicted to be pathogenic by at least two of the in-silico prediction tools. Parental testing showed the heterozygous variant to be de novo, confirmed by X-linked dominant inheritance. Eventually, a diagnosis of MRX102 was made.

Written informed consent was obtained from the family for this study.

## Discussion and conclusions

The clinical diagnosis of CP is based on neurological symptoms and a motor disorder causing an activity limitation. It is reported that 10 risk factors associated with CP: placental abnormalities, birth defects, low birthweight, meconium aspiration, emergency caesarean section, birth asphyxia, neonatal seizures, respiratory distress syndrome, hypoglycaemia, and neonatal infections [15]. Meanwhile, MRI findings are very important in the description of CP. Among the 86% CP patients, periventricular white matter injury is the most frequent finding which accounted for 56%, deep grey matter injury 18%, and brain maldevelopment 9% [16]. Delayed myelination, dysplasia of the corpus callosum, and enlargement of the lateral ventricles also can be found in CP [17, 18].

We report the case of a girl who was considered to have ataxic CP following magnetic resonance imaging revealing areas of cerebral dysplasia at early phase by her primary pediatrician. A primary diagnosis was made according to the presence of cerebral dysplasia, delayed motor development, a non-progressive course, and ataxia combined with frequent falls. However, the presence of her intellectual disability had only been treated as a comorbidity of CP. After a review by a geneticist, a novel variant in the DDX3X gene was identified by the whole-exome sequencing. The variant locus was at the same amino acid residue but with a different pathogenic base pair in the *DDX3X* gene and had been reported before [19]. An American female described previously to also be suffering from unexplained intellectual disability was carrying the *DDX3X* variant: c.1250 A > C, p.Gln417Pro. However, further details were not described [19]. Nearly all published *DDX3X* variants resulting in MRX102 so far are de novo and are proved “damaging in biology” [9]. MRX102 has not been previously confused with ataxic CP until now. This illustrates that in this case, similar characteristics in both disorders should be paid due attention. Additionally, the abnormal MRI results of the patient made the diagnosis difficult in MRX102. Our study makes the importance of the correct diagnosis clear, especially on terms of reproductive genetic counselling for family members.

In summary, the patient reported in this paper exemplifies the problem that clinicians may face in distinguishing between CP and other similar disorders. When a diagnosis is carried out early in childhood, the clinical features may meet the characteristics of CP but a careful review before a final diagnosis is essential. Since there are few reports of this novel *DDX3X* variant, further research into its biological significance and mechanism of action is warranted. The application of whole-exome sequencing in clinical practice among individuals with CP will play a great role in its diagnosis compared with other methods.

## Data Availability

The datasets used and/or analyzed during the current study are available from the corresponding author on reasonable request.

## Abbreviations

CP: Cerebral palsy
*DDX3X*: DEAD/H Box 3, X-LINKED
MRX102: Mental retardation, X-linked 102
MRI: Magnetic resonance imaging
ExAC: Exome Aggregation Consortium database

## References

[1] Garuti G, Verucchi E, Fanelli I, Giovannini M, Winck JC, Lusuardi M (2016). Management of bronchial secretions with free aspire in children with cerebral palsy: impact on clinical outcomes and healthcare resources. Ital J Pediatr.

[2] Bulekbayeva S, Daribayev Z, Ospanova S. Cerebral palsy: a multidisciplinary, integrated approach is essential. Lancet Glob Health. 2017;5(4):e401. 10.1016/S2214-109X(17)30082-7 28288745

[3] Graham HK, Rosenbaum P, Paneth N, Dan B, Lin JP, Damiano DL (2016). Cerebral palsy. Nat Rev Dis Primers.

[4] McMichael G, Bainbridge MN, Haan E, Corbett M, Gardner A, Thompson S (2015). Whole-exome sequencing points to considerable genetic heterogeneity of cerebral palsy. Mol Psychiatry.

[5] Andersen EW, Leventer RJ, Reddihough DS, Davis MR, Ryan MM (2016). Cerebral palsy is not a diagnosis: a case report of a novel atlastin-1 mutation. J Paediatr Child Health.

[6] Appleton RE, Gupta R (2019). Cerebral palsy: not always what it seems. Arch Dis Child.

[7] Ashwal S, Russman BS, Blasco PA, Miller G, Sandler A, Shevell M (2004). Practice parameter: diagnostic assessment of the child with cerebral palsy: report of the quality standards Subcommittee of the American Academy of neurology and the practice Committee of the Child Neurology Society. Neurology..

[8] McMichael G, Haan E, Gardner A, Yap TY, Thompson S, Ouvrier R (2013). NKX2-1 mutation in a family diagnosed with ataxic dyskinetic cerebral palsy. Eur J Med Genet.

[9] Nicola P, Blackburn PR, Rasmussen KJ, Bertsch NL, Klee EW, Hasadsri L (2019). De novo DDX3X missense variants in males appear viable and contribute to syndromic intellectual disability. Am J Med Genet A.

[10] Brennan R, Haap-Hoff A, Gu L, Gautier V, Long A, Schroder M (2018). Investigating nucleo-cytoplasmic shuttling of the human DEAD-box helicase DDX3. Eur J Cell Biol.

[11] Abdelhaleem M (2005). RNA helicases: regulators of differentiation. Clin Biochem.

[12] Cruciat CM, Dolde C, de Groot RE, Ohkawara B, Reinhard C, Korswagen HC (2013). RNA helicase DDX3 is a regulatory subunit of casein kinase 1 in Wnt-beta-catenin signaling. Science..

[13] Dikow N, Granzow M, Graul-Neumann LM, Karch S, Hinderhofer K, Paramasivam N (2017). DDX3X mutations in two girls with a phenotype overlapping Toriello-Carey syndrome. Am J Med Genet A.

[14] Kellaris G, Khan K, Baig SM, Tsai IC, Zamora FM, Ruggieri P (2018). A hypomorphic inherited pathogenic variant in DDX3X causes male intellectual disability with additional neurodevelopmental and neurodegenerative features. Hum Genomics.

[15] Wimalasundera N, Stevenson VL (2016). Cerebral palsy. Pract Neurol.

[16] Himmelmann K, Horber V, De La Cruz J, Horridge K, Mejaski-Bosnjak V, Hollody K (2017). MRI classification system (MRICS) for children with cerebral palsy: development, reliability, and recommendations. Dev Med Child Neurol.

[17] Ali A, Yalçın R, Ünlüer-Gümüştaş A (2019). Cranial MR characteristics of cerebral palsy cases and correlation of findings with clinical results. Turk J Pediatr.

[18] Seidl Z, Süssová J, Obenberger J, Vanecková M, Viták T, Rydland J (2001). Magnetic resonance imaging in diplegic form of cerebral palsy. Brain Dev.

[19] Snijders Blok L, Madsen E, Juusola J, Gilissen C, Baralle D, Reijnders MR (2015). Mutations in DDX3X are a common cause of unexplained intellectual disability with gender-specific effects on Wnt signaling. Am J Hum Genet.

